# The Student's Handbook of Surgical Operations

**Published:** 1893-03

**Authors:** 


*The Student's Handbook of Surgical Operations.* By FREDERICK TREVES, F.R.C.S. Pp. xv., 487. London: CASSELL & COMPANY, LIMITED. 1892.

This is a small book abridged from the author's *Manual of Operative Surgery*, and is intended as a handbook for students preparing for the final examinations. For this purpose it cannot fail to prove of service, as it contains a great deal of information of the best kind. The larger work is so good and complete that it seems in many respects a pity to spoil it by boiling it down into such a small compass as this; but we suppose that students must have small books, and we can only wish that they were all so good as the one before us. The descriptions are pithy, the diagrams are clear, and the author has spared no pains to bring his information well up to date. We can recommend it to all who cannot afford the "Manual," or who wish for a brief epitome to use in the *post-mortem* room.

